# Development and validation of a modified quick SOFA scale for risk assessment in sepsis syndrome

**DOI:** 10.1371/journal.pone.0204608

**Published:** 2018-09-26

**Authors:** Yasemin Cag, Oguz Karabay, Oguz Resat Sipahi, Firdevs Aksoy, Gul Durmus, Ayse Batirel, Oznur Ak, Zeliha Kocak-Tufan, Aynur Atilla, Nihal Piskin, Turkay Akbas, Serpil Erol, Derya Ozturk-Engin, Hulya Caskurlu, Ugur Onal, Haluk Erdogan, Aslıhan Demirel, Arzu Dogru, Rezan Harman, Aziz Ahmad Hamidi, Derya Karasu, Fatime Korkmaz, Pınar Korkmaz, Fatma Civelek Eser, Yalcin Onem, Sinem Cesur, Musa Salmanoglu, İlknur Erdem, Hüsrev Diktas, Haluk Vahaboglu

**Affiliations:** 1 Istanbul Medeniyet Universitesi Tip Fakultesi, Enfeksiyon Hastaliklari ve Klinik Mikrobiyoloji Department, Istanbul, Turkey; 2 Sakarya Universitesi Tip Fakultesi, Enfeksiyon Hastaliklari ve Klinik Mikrobiyoloji Department, Sakarya, Turkey; 3 Ege Universitesi Tip Fakultesi, Enfeksiyon Hastaliklari ve Klinik Mikrobiyoloji Department, Izmir, Turkey; 4 Karadeniz Teknik Universitesi Tip Fakultesi, Enfeksiyon Hastaliklari ve Klinik Mikrobiyoloji Department, Trabzon, Turkey; 5 Bursa Sevket Yilmaz Egitim ve Arastirma Hastanesi, Enfeksiyon Hastaliklari ve Klinik Mikrobiyoloji Department, Bursa, Turkey; 6 Dr. Lutfi Kirdar Kartal Egitim ve Arastirma Hastanesi, Enfeksiyon Hastaliklari ve Klinik Mikrobiyoloji Department, Istanbul, Turkey; 7 Yıldırım Beyazıt Universitesi, Ankara Ataturk Egitim ve Arastirma Hastanesi, Enfeksiyon Hastaliklari ve Klinik Mikrobiyoloji Department, Ankara, Turkey; 8 Samsun Egitim ve Arastirma Hastanesi, Enfeksiyon Hastaliklari ve Klinik Mikrobiyoloji Department, Samsun, Turkey; 9 Bulent Ecevit Universitesi Tip Fakultesi, Enfeksiyon Hastaliklari ve Klinik Mikrobiyoloji Department, Zonguldak, Turkey; 10 Duzce Universitesi Tip Fakultesi, İc Hastaliklari Department, Yogun Bakım Section, Istanbul, Turkey; 11 Haydarpasa Numune Egitim ve Arastirma Hastanesi, Enfeksiyon Hastaliklari ve Klinik Mikrobiyoloji Department, Istanbul, Turkey; 12 Baskent Universitesi, Alanya Egitim ve Arastirma Hastanesi, Enfeksiyon Hastaliklari ve Klinik Mikrobiyoloji Department, Antalya, Turkey; 13 Istanbul Bilim Universitesi Tip Fakultesi, Enfeksiyon Hastaliklari ve Klinik Mikrobiyoloji Department, Istanbul, Turkey; 14 Ozel Sani Konukoglu Hastanesi, Gaziantep, Turkey; 15 Şişli Hamidiye Etfal Egitim ve Arastirma Hastanesi, Enfeksiyon Hastaliklari ve Klinik Mikrobiyoloji Department, Istanbul, Turkey; 16 Bursa Sevket Yilmaz Egitim ve Arastirma Hastanesi, Anestezi ve Reanimasyon Department, Bursa, Turkey; 17 Konya Egitim ve Arastirma Hastanesi, Enfeksiyon Hastaliklari ve Klinik Mikrobiyoloji Department, Konya, Turkey; 18 Dumlupinar Universitesi Tip Fakultesi, Enfeksiyon Hastaliklari ve Klinik Mikrobiyoloji Department, Kutahya, Turkey; 19 Ankara Diskapı Yildirim Beyazit Egitim ve Arastirma Hastanesi, Enfeksiyon Hastaliklari ve Klinik Mikrobiyoloji Department, Ankara, Turkey; 20 Sultan Abdulhamid Han Egitim ve Arastirma Hastanesi, İc Hastaliklari Department, Istanbul, Turkey; 21 Namık Kemal Universitesi Tip Fakultesi, Enfeksiyon Hastaliklari ve Klinik Mikrobiyoloji Department, Tekirdag, Turkey; 22 Tatvan Military Hospital, Enfeksiyon Hastaliklari ve Klinik Mikrobiyoloji Department, Van, Turkey; Azienda Ospedaliero Universitaria Careggi, ITALY

## Abstract

Sepsis is a severe clinical syndrome owing to its high mortality. Quick Sequential Organ Failure Assessment (qSOFA) score has been proposed for the prediction of fatal outcomes in sepsis syndrome in emergency departments. Due to the low predictive performance of the qSOFA score, we propose a modification to the score by adding age. We conducted a multicenter, retrospective cohort study among regional referral centers from various regions of the country. Participants recruited data of patients admitted to emergency departments and obtained a diagnosis of sepsis syndrome. Crude in-hospital mortality was the primary endpoint. A generalized mixed-effects model with random intercepts produced estimates for adverse outcomes. Model-based recursive partitioning demonstrated the effects and thresholds of significant covariates. Scores were internally validated. The *H* measure compared performances of scores. A total of 580 patients from 22 centers were included for further analysis. Stages of sepsis, age, time to antibiotics, and administration of carbapenem for empirical treatment were entered the final model. Among these, severe sepsis (OR, 4.40; CIs, 2.35–8.21), septic shock (OR, 8.78; CIs, 4.37–17.66), age (OR, 1.03; CIs, 1.02–1.05) and time to antibiotics (OR, 1.05; CIs, 1.01–1.10) were significantly associated with fatal outcomes. A decision tree demonstrated the thresholds for age. We modified the quick Sequential Organ Failure Assessment (mod-qSOFA) score by adding age (> 50 years old = one point) and compared this to the conventional score. *H*-measures for qSOFA and mod-qSOFA were found to be 0.11 and 0.14, respectively, whereas AUCs of both scores were 0.64. We propose the use of the modified qSOFA score for early risk assessment among sepsis patients for improved triage and management of this fatal syndrome.

## Introduction

Despite the growing accessibility to effective antibiotics in medical practice, sepsis syndrome is still a serious infectious disease with high morbidity and mortality [1]. Sepsis syndrome consisted of three sequential stages: sepsis, severe sepsis, and septic shock [2]. Sepsis was defined as having two or more of the Systemic Inflammatory Response Syndrome (SIRS) criteria attributed to a suspected or documented infection [2]. If sepsis was misdiagnosed or unnoticed for any reason, severe sepsis and septic shock develop consecutively. Studies have shown that mortality rates increase serially in sepsis, severe sepsis, and septic shock from 10% to 80% [3].

SIRS based sepsis definition is broad and nonspecific which causes a considerable number of false diagnosis [4,5]. Recently, a task force proposed new sepsis definitions which were grounded on the SOFA or qSOFA scores (≥ 2 points) [6]. Early studies indicated that the predictive performance of sepsis-3 definitions was superior to SIRS based definitions in predicting adverse outcomes [7].

Using qSOFA score in emergency departments (ED) seems to be a practical substitute to stratify patients with infection. However, the debate on the performance of sepsis-3 definitions are ongoing [8] and, in recent studies, sepsis-3 definitions yielded poor sensitivity for predicting adverse outcomes [9]. Refining the predictive performance of qSOFA with a reasonable trade-off between misclassification types is of interest.

In this study, we studied the marginal effects of risk variables associated with adverse outcomes and based on our estimates we developed a modification to improve the cost-sensitive predictive performance of qSOFA in sepsis.

## Materials and methods

### Study design

We performed a multi-center, retrospective cohort study among adult patients admitted to the emergency services of regional referral hospitals with a diagnosis of sepsis syndrome from March 2013 to January 2016.

The Ethical Committee of Istanbul Medeniyet University (Istanbul, Turkey) approved this study with a waiver of informed consent (#2015/0142).

### Study setting, population, and data collection

Referral centers from various regions of Turkey participated in the study. At least two physicians specializing in related fields extracted potential cases from the hospital databases using sepsis and SIRS-related ICD-10 codes. Participants screened medical records for the eligibility of patients according to study inclusion and exclusion criteria. Finally, researchers extracted data from medical charts of patients and the hospital records. The centers submitted their data on a spreadsheet.

### Definitions

Study inclusion criteria were as follows: i. Age > 17 years old; ii. Outpatient with a diagnosis of sepsis syndrome upon admission to the emergency department (ED). Patients with incomplete outcome information and those with sepsis that developed in the hospital after being admitted for other medical reasons were excluded from the analysis.

Sepsis was defined as having two or more of SIRS criteria attributed to a suspected or documented infection. Severe sepsis was defined as sepsis plus organ dysfunction attributed to sepsis-induced tissue hypoperfusion. Septic shock was defined as severe sepsis plus refractory hypotension despite adequate fluid resuscitation [10]. Organ dysfunctions were as defined elsewhere [10].

Elapsed time to antibiotics was the elapsed time between ED admission and the administration of antibiotics. Underlying diseases were grouped according to principal component analysis. Accordingly, patients were coded as positive for underlying diseases, including any of diabetes, chronic renal disease, and solid organ malignancies.

Data on age, gender, blood culture results, carbapenem administration as initial treatment approach, intensive care unit (ICU) stay during the course of the disease, mechanical ventilation in ICU, suspected source of infection upon admission, leucocyte count upon admission, length of hospital stay (days), and SOFA score were also collected.

The outcome of interest was in-hospital crude mortality.

We performed a post-hoc modification of the qSOFA score by including age as the fourth parameter. Eventually, we compared the predictive performance for the outcome of the mod-qSOFA score against the classical qSOFA.

### Statistical analysis

Data management and statistical analysis were performed using the open-source statistical packages on R (a language and environment for statistical computing, R Foundation for Statistical Computing, Vienna, Austria. URL: https://www.R-project.org).

Continuous variables if non-normally distributed were presented as median, first and third quartiles and compared using the Kruskal-Wallis test in a univariate analysis. Otherwise, continuous variables were presented as means and standard deviations and compared using Students t-test. Categorical variables were compared using chi-squared or, where required, Fisher’s exact test.

We imputed missing values after testing the missing mechanisms of variables for missing completely at random (MCAR) [11]. Missing variables were imputed 20 times by multivariate imputations with chained equations by using the best-suited method [12]. Estimates were pooled according to Rubin’s rules. We also generated a complete data set by aggregating twenty imputations to medians. Since the estimates from the aggregated data set are almost identical to the pooled estimates, we used the aggregated single data set for further analysis.

To estimate the effects of the variables, we fitted a generalized linear mixed-effects model with random intercepts. We selected the final model using the least absolute shrinkage and selection operator (LASSO) without random effects [13] and Akaike’s information criterion (AIC/BIC) with random effects among all potential risk variables. We tested the multicollinearity between risk variables, and if it exists, we avoided collinearity by dropping the responsible variable from the model. Interactions between the covariates of the final model were examined one by one to each other.

To assess thresholds of significant predictors, we applied a generalized linear model-based recursive partitioning by the “glmtree” function of the “partykit” package. The details of this approach have been published [14].

We fragmented the database randomly to train and test subsets with a ratio of 0.5. Scores were trained via linear discriminant analysis and tested by using the “hmeasure” package for their classifying performances as described elsewhere [15]. We compared performances of scores by *H* measure and area under Receiver Operating Characteristic (ROC) curve (AUC). AUC estimates performances of scores by giving equal weights to the false negatives and false positives whereas *H* measure enables to select a risk ratio according to the relative severities of types of misclassification.

## Results

A total of 22 referral centers recruited data from 660 eligible patients. Among these, 80 patients were excluded from the analysis due to incomplete outcome data. These outcome data were missing because those patients were i) staying in the hospital during the study, ii) transferred to another hospital, or iii) the outcome data were not recorded into the hospital database. In total, 580 patients were included in the analysis (S1 Table).

Table 1 presents the demographics and other features of the study population. Briefly, the median age was 73 years old, and the male to female ratio was comparable. Two-thirds of the patients (68.1%) admitted to the EDs at the early, mild stage of sepsis syndrome. A total of 32.6% of the patients died in the hospital.

**Table 1:** Demographics and other features of the cohort.

	N = 580	N
Age	73 [59;81]	579
Gender:		580
Female	250 (43.1%)	
Male	330 (56.9%)	
Blood culture positive	194 (33.4%)	580
Carbapenem ^a^	154 (26.6%)	580
ICU stay	295 (50.9%)	580
Mechanical ventilation	186 (32.7%)	568
Elapsed time (hours) ^b^	3 [2; 5]	533
Underlying diseases ^c^		580
No	296 (51.0%)	
Yes	284 (49.0%)	
Suspected source		580
Urinary tract	236 (40.7%)	
Lower respiratory tract	151 (26.0%)	
Skin-soft tissue	33 (5.69%)	
Intra-abdominal	30 (5.17%)	
Catheter related	18 (3.10%)	
Other	61 (10.5%)	
Unknown	51 (8.79%)	
Leucocyte (mm^3)	14000 [9792;19500]	580
Length of hospital stay (days)	13.0 [8.00;18.0]	580
Stage of sepsis		580
Sepsis	395 (68.1%)	
Severe sepsis	112 (19.3%)	
Septic shock	73 (12.6%)	
SOFA score	4 [2;8]	505
Died:		580
No	391 (67.4%)	
Yes	189 (32.6%)	

^**a**^ Carbapenem, a carbapenem antibiotic was instituted at admission

^**b**^ Elapsed time, time between application to ED and administration of an antibiotic

^**c**^ Underling diseases, having any of diabetes, renal insufficiency or a malign disease (Underlying diseases are presented individually in S2 Table).

Although all the centers obtained blood cultures timely using modern blood culture systems, microbiology laboratories were able to recover a microorganism only from one-third of the samples (33.4%). *E*. *coli* was the most common bacteria (43.8% of all isolated bacteria) followed by *S*. *aureus* (18.6%), coagulase-negative *Staphylococcus* (9.7%), *K*. *pneumoniae* (7.7%), *Enterococcus* spp (5%), *Enterobacteriaceae* (4.1%), and *S*. *pneumoniae* (4.1%).

Almost one-fifth of the suspected sources of infections reported by the participants were either unknown or not classified under major sites. Since this parameter is highly speculative, and such a large portion could not be classified in our study, we did not include the suspected source of infection in the analysis. However, a suspected source of lower respiratory tract infection seems to be associated with adverse outcomes (data not shown). In a carefully designed prospective study, this variable could be effectively examined to further improve discrimination ability.

Three of the examined variables had missing observations. Age had one, elapsed time to antibiotics had 47, and qSOFA score had 75 missing observations. The hypothesis of MCAR was rejected at the 0.05 level by the normality test; therefore, dropping the missing observations would produce biased estimates. We imputed the missing observations 20 times, thereby generating twenty different datasets. We also generated a complete data set that by aggregating the set of twenty imputations to the medians.

Table 2 presents the univariate comparisons of the risk variables. The potential risk variables found to be significant from this comparison included: age, having a negative blood culture, elapsed time to antibiotics, and stage of sepsis. However, we included all potential variables into the automatic variable selection routine. Age, elapsed time to antibiotics, stage of sepsis, and empirical carbapenem usage were selected for the final model. Blood culture was identified as a significant variable from the univariate analysis. However, the AIC/BIC-based model selection excluded this variable due to its minimal contribution to the discrimination power of the final model.

**Table 2:** Univariate comparison of risk variables.

	Died		*p*
	No N = 391	Yes N = 189	
Age	69 [55;80]	78 [69;84]	<0.001
Gender			0.995
Female	168 (67.2%)	82 (32.8%)	
Male	223 (67.6%)	107 (32.4%)	
Blood culture			0.006
Negative	245 (63.5%)	141 (36.5%)	
Positive	146 (75.3%)	48 (24.7%)	
Carbapenem ^a^			0.254
No	281 (66.0%)	145 (34.0%)	
Yes	110 (71.4%)	44 (28.6%)	
Elapsed time (hours) ^b^	2 [2;4]	4 [2;6]	<0.001
Underlying diseases ^c^	185 (65.1%)	99 (34.9%)	0.291
Stage of sepsis			<0.001
Sepsis	312 (79.0%)	83 (21.0%)	
Severe sepsis	59 (52.7%)	53 (47.3%)	
Septic shock	20 (27.4%)	53 (72.6%)	

^**a**^ Carbapenem, a carbapenem antibiotic is instituted at admission

^**b**^ Elapsed time, the time between application to ED and administration of an antibiotic

^**c**^ Underling diseases, having any of diabetes, renal insufficiency or a malign disease

Table 3 presents the estimates from the mixed model with random intercepts for centers. Briefly, the random part of the summary table shows that the intraclass correlation between centers is high (ICCcenter, 0.323), which intends the need to account for random effects. The fixed part of the summary table shows the association between stages of sepsis, age, and elapsed time to antibiotics with fatal outcomes.

**Table 3:** Estimates from generalized mixed models with random intercepts.

	Single estimates ^a^			Pooled estimates ^b^		
	*Odds Ratio*	*CI* ^c^	*p*	*Odds Ratio*	*CI*	*p*
**Fixed Parts**						
(Intercept)	0.02	0.00–0.07	<0.001	0.02	0.01–0.10	< 0.001
Severe sepsis	4.40	2.35–8.21	<0.001	4.43	2.35–8.34	< 0.001
Septic shock	8.78	4.37–17.66	<0.001	9.18	4.54–18.50	< 0.001
Age	1.03	1.02–1.05	<0.001	1.03	1.01–1.05	0.002
Elapsed time ^d^	1.05	1.01–1.10	.018	1.05	1.00–1.10	0.033
Carbapenem ^e^	0.64	0.37–1.11	.112	0.68	0.39–1.18	0.17
**Random Parts** ^f^						
τ_00, Center_	1.566					
N_Center_	22					
ICC_Center_	0.323					
Observations	580					
Deviance	466.753					

^**a**^ Single estimates, estimates obtained from the aggregated data set,

^**b**^ Pooled estimates, pooled estimates according to Rubin’s rule

^**c**^ CI, 95% confidence intervals

^**d**^ Elapsed time, the time between application to ED and administration of an antibiotic

^**e**^ Carbapenem, a carbapenem antibiotic is instituted at admission

^**f**^ τ_00, Center_, the variance of random intercept; ICC_center_; intraclass correlation coefficient (“0” indicates that between centers effect is negligible)

**Fig 1:**
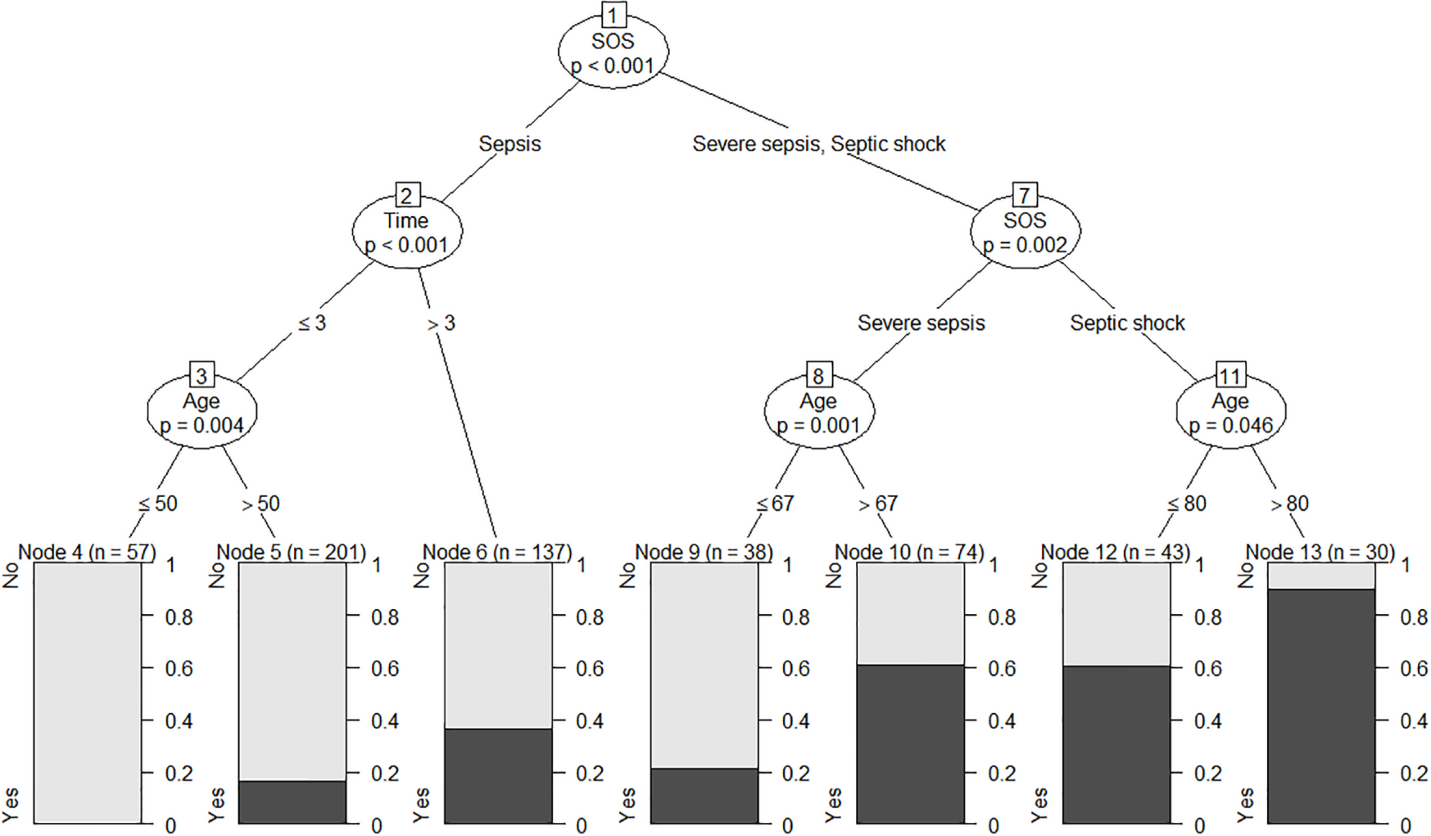
The model-based decision tree for fatal outcomes among patients with sepsis syndrome. The fatal outcome is first partitioned among stages of sepsis (SOS). Sepsis node partitioned by time to antibiotics, followed by age. Age partitioned severe sepsis and septic shock nodes. Terminal nodes displayed as bar plots giving the percentages of fatal outcomes in the node. Of notice was the patients under 50 years old who received antibiotics within three hours were all survived.

Fig 1 represents the model-based decision tree for fatal outcomes. The relationship between fatal outcome and elapsed time to antibiotics, age, and stage of sepsis is presented. Briefly, all 57 patients admitted to the ED with a diagnosis of sepsis, who were equal or younger than 50 years old and received antibiotics within three hours survived. Hence, we selected 50 years as the threshold for age. The modified qSOFA (mod-qSOFA) adds one point for age > 50 years. Positive test results were ≥ 2 points, as was in the traditional scale.

**Fig 2:**
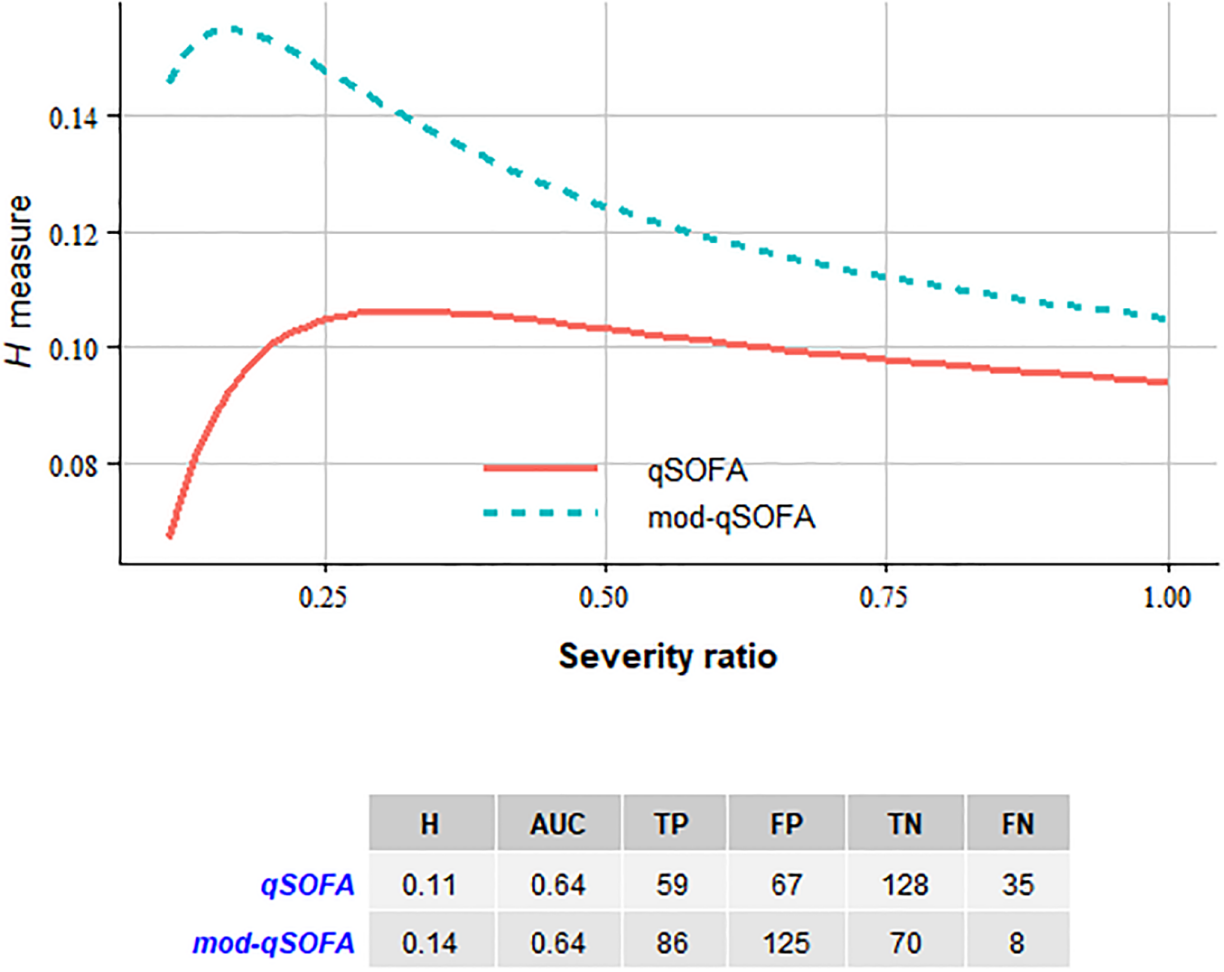
Comparative performances of scores. (A) *H* measure at different severity ratios. Severity ratio is the ratio of the cost of false positive over the cost of false negative predictions.; (B) Table of *H* measure, AUC, true positive (TP), false positive (FP), true negative (TN) and false negative (FN) predictions of scores.

Fig 2 presents comparisons of predictive performances of scores. Briefly, *H* measure favors the predictive performance of mod-qSOFA across various risk ratios. Values given in the table is at the severity ratio of 0.25 which means that the cost of misclassifying a potentially non-fatal case is 0.20 and a fatal case is 1 minus 0.20 (SR = c/1-c). AUC’s of both scores were equal. SOFA score misclassified 35 fatal patients whereas mod-qSOFA misclassified only eight fatal patients at an exchange of 58 non-fatal cases (FP) in the test subset (n = 289) of the data.

## Discussion

In this study we developed and validated the mod-qSOFA score against the traditional version using various metrics such as AUC and *H* measure. In the internal validation, the mod-qSOFA score yielded better performance with the *H* measure metrics.

The AUC values were comparable between scores. However, AUC is not a valid metric for evaluating scores in sepsis syndrome. In a medical situation with high mortality like sepsis syndrome, the performance of a classifier should be evaluated with cost-sensitive statistics. The cost of misclassifying a potentially fatal case is not equivalent to the cost of misclassifying a nonfatal case. In sepsis syndrome, the cost of a false negative (FN) is greater than the cost of a false positive (FP). The AUC weights costs of FPs and FNs in a balanced fashion. AUC of ROC, thus, is not suitable to measure the performances of classifiers in sepsis syndrome. The *H* measure allows to manually adjust the risk ratio according to which type of misclassification is more serious. Risk ratio is the ratio of cost of FP over the cost of FN. The risk ratio is selected arbitrarily, largely depending on the expertise. To estimate systematically, thus, we presented the distribution of *H* measure values obtained across various risk ratios (Fig 2). However, an optimum risk ratio that makes sense for sepsis syndrome might be 0.25. A risk ratio of 0.25 means that the cost of false alarm is 0.2 whereas the cost of missing a fatal case is 0.8.

In our study, using mod-qSOFA reduced the percentage of false negative decisions by 9% (35-8/289) compare to the traditional qSOFA. In clinical practice, correctly identifying the subset of patients with potentially adverse clinical outcomes is of major importance.

Our study documented how the patient outcome was affected in different stages of sepsis syndrome by age and time to antibiotic treatment. Overall, in all the stages of sepsis syndrome, older ages were more associated with adverse outcomes. The relation between age and adverse outcomes was connected to the frequency of comorbidities among elderly populations [16]. With high comorbidities, older patients have a high risk of adverse outcomes. Studies using various cutoff points found a strong association between age and fatal outcomes [16,17]. In all these studies, however, mortality augmented dramatically after the fifties. In our study, we also noticed a similar effect of age on the outcome (Fig 1). Depending on our results, we, propose adding age (> 50 years old = one point) as a fourth parameter to the qSOFA score. In our database, this modification increased the predictive performance of the qSOFA score on the outcome.

Our study also documented that elapsed time to antibiotic treatment was independently associated with adverse outcomes, especially in the early stages of sepsis syndrome. Depending on the elapsed time before admittance, awareness of the physician and the adequacy of the empirical antibiotics, time to antibiotics in EDs might be highly variable [18]. Hence, we did not consider to include time to antibiotics in mod-qSOFA.

A quarter of the patients in this study received the antibiotic carbapenem initially. Recent reports from Turkey reveal the high prevalence of extended-spectrum beta-lactamase production among community-acquired *E*. *coli* infections [19]. This upward trend in resistance frequency among the most common cause of community-acquired sepsis might partially explain the high number of carbapenem prescriptions upon admission.

In this study, we chose not to include an administrative time censor for mortality (e.g. 28-day mortality). To set an administrative censor would require the use of a “time-to-event” analysis. In our opinion, such a time-to-event analysis would not contribute appreciably to the main purpose of this study, which was to develop an improved scoring system.

The major weakness of this study is its retrospective design, which represents a source of potential selection bias, especially regarding age. Another limitation is the relatively small sample size in this study, which weakens the generalizability of the findings. We, therefore, suggest validating the predictive performance of the mod-qSOFA score in prospective studies. One other limitation is the high number of negative blood culture results. Because of the high proportion of negative blood cultures, we could not adjust our estimates with such a significant confounder. However, the poor sensitivity of blood cultures in community-acquired sepsis syndrome is quite ordinary [20]. We believe that adjusting a multivariate model by the adequacy of antibiotic treatment using a minor subset of the cohort (a subset with positive blood cultures) would generate biased estimates.

## Conclusions

We propose the implementation of the mod-qSOFA in EDs toward improving early identification of high-risk patients with sepsis syndrome along with other measures. Adopting this modified assessment system may improve patient stratification, facilitate appropriate allocation of resources, and optimize patient care.

## Supporting information

S1 Table Contributing centers and patient numbers. **^a^** Centers were coded and names were hidden.
(DOCX)

S2 Table Underlying diseases in the study population. (DOCX)

S1 Dataset Database. (ZIP)
