# Supplementary material for: Development and validation of a modified quick SOFA scale for risk assessment in sepsis syndrome
Source: PLoS One. 2018 Sep 26;13(9):e0204608. doi: 10.1371/journal.pone.0204608 (PMC6157867; doi:10.1371/journal.pone.0204608)
Supplement: S1 Table — a Centers were coded and names were hidden. (DOCX) [file pone.0204608.s001.docx]

**S1 Table. Contributing centers and patient numbers**

|  | Center ^[[1]](#footnote-1)^ | n |
| --- | --- | --- |
| 1 | 2 | 20 |
| 2 | 3 | 22 |
| 3 | 4 | 20 |
| 4 | 5 | 52 |
| 5 | 7 | 110 |
| 6 | 8 | 18 |
| 7 | 9 | 20 |
| 8 | 10 | 25 |
| 9 | 11 | 20 |
| 10 | 12 | 29 |
| 11 | 13 | 3 |
| 12 | 14 | 7 |
| 13 | 15 | 37 |
| 14 | 16 | 19 |
| 15 | 17 | 20 |
| 16 | 18 | 30 |
| 17 | 19 | 18 |
| 18 | 20 | 17 |
| 19 | 21 | 33 |
| 20 | 22 | 38 |
| 21 | 23 | 19 |
| 22 | 24 | 3 |

1. Centers were coded and names were hidden [↑](#footnote-ref-1)
