# Supplementary material for: Development and validation of a modified quick SOFA scale for risk assessment in sepsis syndrome
Source: PLoS One. 2018 Sep 26;13(9):e0204608. doi: 10.1371/journal.pone.0204608 (PMC6157867; doi:10.1371/journal.pone.0204608)
Supplement: S2 Table — (DOCX) [file pone.0204608.s002.docx]

**S2 Table. Underlying diseases in the study population**

|  | N (%) | N |
| --- | --- | --- |
| Diabetes mellitus | 172 (29.7%) | 580 |
| Chronic renal insufficiency | 102 (17.6%) | 580 |
| Chronic obstructive pulmonary disease | 84 (14.5%) | 580 |
| Cerebrovascular accident | 142 (24.5%) | 580 |
| Solid organ malignancy | 76 (13.1%) | 578 |
| Hematologic malignancy | 17 (2.94%) | 579 |
| Coronary syndrome | 129 (22.3%) | 579 |
| Congestive heart disease | 84 (14.5%) | 579 |
| Auto-inflammatory/immune disease | 5 (0.86%) | 580 |
